# Prolonged ozone exposure in an allergic airway disease model: Adaptation of airway responsiveness and airway remodeling

**DOI:** 10.1186/1465-9921-7-24

**Published:** 2006-02-13

**Authors:** An-Soo Jang, Inseon-S Choi, Jae-Hyuk Lee, Chang-Soo Park, Choon-Sik Park

**Affiliations:** 1 Department of Internal Medicine, Chonnam National University Medical School, Gwangju, Republic of Korea; 2 Pathology, Chonnam National University Medical School, Gwangju, Republic of Korea; 3 Department of Internal Medicine, Soonchunhyang University Hospital, Bucheon, Gwangju, Republic of Korea

## Abstract

**Background:**

Short-term exposure to high concentrations of ozone has been shown to increase airway hyper-responsiveness (AHR). Because the changes in AHR and airway inflammation and structure after chronic ozone exposure need to be determined, the goal of this study was to investigate these effects in a murine model of allergic airway disease.

**Methods:**

We exposed BALB/c mice to 2 ppm ozone for 4, 8, and 12 weeks. We measured the enhanced pause (Penh) to methacholine and performed cell differentials in bronchoalveolar lavage fluid. We quantified the levels of IL-4 and IFN-γ in the supernatants of the bronchoalveolar lavage fluids using enzyme immunoassays, and examined the airway architecture under light and electron microscopy.

**Results:**

The groups exposed to ozone for 4, 8, and 12 weeks demonstrated decreased Penh at methacholine concentrations of 12.5, 25, and 50 mg/ml, with a dose-response curve to the right of that for the filtered-air group. Neutrophils and eosinophils increased in the group exposed to ozone for 4 weeks compared to those in the filtered-air group. The ratio of IL-4 to INF-γ increased significantly after exposure to ozone for 8 and 12 weeks compared to the ratio for the filtered-air group. The numbers of goblet cells, myofibroblasts, and smooth muscle cells showed time-dependent increases in lung tissue sections from the groups exposed to ozone for 4, 8, and 12 weeks.

**Conclusion:**

These findings demonstrate that the increase in AHR associated with the allergic airway does not persist during chronic ozone exposure, indicating that airway remodeling and adaptation following repeated exposure to air pollutants can provide protection against AHR.

## Introduction

Asthma is characterized by the presence of a variable airflow limitation, airway hyper-responsiveness (AHR), and airway inflammation [1]. Acute exposure to ozone, which is an important component of the photochemical oxidation products of substrates emitted as air pollution from automobile engines [2], decreases pulmonary function, increases AHR, and induces airway inflammation in dogs [3], guinea pigs [4], and humans [5-7]. Chronic airway inflammation is associated with airway remodeling that includes airway wall thickening as a result of inflammatory and structural changes, such as edema; inflammatory cell infiltration; mucous gland hyperplasia; reticular basement membrane thickening; subepithelial fibrosis; vascular smooth muscle cell proliferation, hyperplasia, and hypertrophy; and myofibroblast and goblet cell hypertrophy [8-11]. Airway wall thickening and airway reactivity were inversely associated in patients with asthma, suggesting that airway wall thickening prevents excessive airway narrowing in human subjects *in vivo *[12].

Interleukin (IL)-4 is key factor contributing to the chronic inflammatory state that characterizes asthma and may be involved in the connective tissue alterations that characterize airway remodeling in asthma. IL-4 can stimulate fibroblasts [13]. Interferon (IFN)-γ, thought to be deficient in asthma, can antagonize some of the effects of IL-4 [14].

The effects of long-term, repeated exposure to ozone on AHR and airway structural changes remain poorly defined. Our underlying hypothesis is that repeated episodes of ozone exposure give rise to some of the remodeling changes associated with asthma, which may in turn be associated with sustained airway dysfunction. The aims of this study were to examine the relationship between ozone exposure and AHR by using barometric whole-body plethysmography (WBP) and to characterize the airway structural changes following a daily 8-h exposure to 2 ppm ozone for 4, 8, and 12 weeks in a murine model of asthma. Airway inflammation was also assessed by analysis of bronchoalveolar lavage (BAL) fluid.

## Methods

### Mice

Female BALB/c mice (aged 5 to 6 weeks; DaeMul Laboratories, Daejeon, Korea) known to be high IgE responders were used. The mice were maintained on an ovalbumin (OVA)-free diet and were individually housed in rack-mounted stainless steel cages with free access to food and water.

### Ovalbumin-induced allergic airway disease model

An OVA-induced allergic airway disease model of asthma was used with some modification [15]. Briefly, mice were sensitized on days 1 and 14 by intraperitoneal injection with 10 μg of grade V OVA (Sigma Chemicals, St. Louis, MO) and 1 mg of aluminum potassium sulfate (Sigma Chemicals) in 500 μL of saline solution. On days 21 to 23, the mice were challenged by daily exposure (30 min) to an aerosol of 1% (wt/vol) OVA in saline solution. Vehicle control mice were treated with a suspension of aluminum potassium sulfate (1 mg) in saline solution (500 μL) and challenged with aerosolized saline solution daily from days 21 to 23. Aerosol challenge was conducted on groups of up to 12 mice in a closed chamber attached to an ultrasonic nebulizer (NE-UO7; Omron Corporation, Tokyo, Japan) with an output of 1 mL/min and 1- to 5-μm particle size.

### Ozone exposure

**Figure 1:**
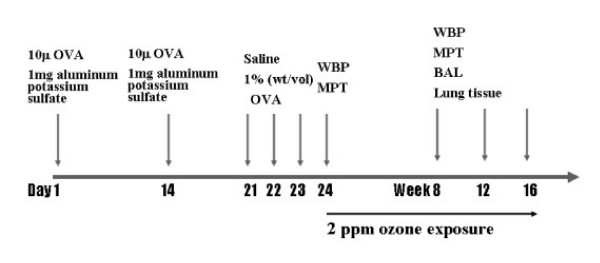
Schematic of the sensitization protocol. Sensitized mice were challenged with 1% (wt/vol) ovalbumin for 30 min on days 21–23. Groups of mice were exposed to 2 ppm ozone for 8 h per day for 4, 8, and 12 weeks, respectively. Whole-body plethysmography was performed at 24 days and at 8, 12, and 16 weeks. Broncholalveolar lavage fluid and lung tissue were obtained at 24 days and at 8, 12, and 16 weeks.

The mice housed in whole-body exposure chambers were exposed to ozone concentrations of 2 ppm for 4, 8, and 12 wks (*n *= 6; Fig. 1); the ozone doses and exposure times were selected based on our previous study [16]. Ozone was generated with Sander model 50 ozonizers (Sander, Eltze, Germany). The concentration of ozone within the chambers was monitored throughout the exposure with ambient-air ozone motors (model 49 C; Thermo Environmental Instruments Inc., Franklin, MA). The air-sampling probes were placed in the breathing zone of the mice. The mean chamber ozone concentration (± SE) during the 8-h exposure period was 1.92 ± 0.15 ppm. The breathing parameter values of spontaneously breathing BALB/c mice were determined under standard conditions at room air and temperature.

### Determination of airway responsiveness

Airway responsiveness was measured by barometric plethysmography using whole-body plethysmography (WBP; Buxco, Troy, NY) after ozone exposure, while the animals were awake and breathing spontaneously as a modification of the method described by Hamelmann et al. [17]. Enhanced pause (Penh) to methacholine as measured using barometric plethysmography is a valid indicator of bronchoconstriction in mice and can be used to measure AHR [17-19]. Aerosolized methacholine in increasing concentrations (2.5–50 mg/ml) was nebulized through an inlet of the main chamber for 3 min.

Bronchoconstriction alters breathing patterns, and changes in the timing of early and late expirations (Pause) and in Penh are the results of alterations in the timing of breathing, as well as the prolongation of the expiratory time. Furthermore, airway constriction increases the thoracic flow asynchronously with the nasal flow, resulting in an increase in the box pressure signal. Penh is an empiric parameter that reflects changes in the waveform of the measured box pressure signal that are a consequence of bronchoconstriction. Before taking readings, the box was calibrated with a rapid injection of 150 μl of air into the main chamber. The difference between the pressure in the main chamber of the WBP containing the animal and that in a reference chamber was measured as the box pressure signal, which is caused by the pressure change in the main chamber during the respiratory cycle of the animal. A pneumotachograph with defined resistance in the wall of the main chamber acted as a low-pass filter and allowed thermal compensation. The time constant of the box was determined to be approximately 0.02 s. Mice were placed in the main chamber, and baseline readings were taken and averaged for 3 min.

### BAL fluid preparation and analysis

BAL was performed immediately after the last measurement of airway responsiveness. The mice were deeply anesthetized with 50 mg/kg of pentobarbital sodium injected intraperitoneally and were killed by exanguination from the abdominal aorta. The trachea was cannulated with a polyethylene tube through which the lungs were lavaged three times with 1.0 ml of physiological saline (4.0 ml total fluid removed). The BAL fluid was filtered through wet gauze (4 × 4 inches). Trypan blue exclusion for viability and total cell count was performed. The BAL fluid was centrifuged at 150 × *g *for 10 min. The obtained pellet was immediately suspended in 4 ml of physiological saline, and total cell numbers in the BAL fluid were counted in duplicate with a hemocytometer (improved Neubauer counting chamber). A 100-μl aliquot was centrifuged in a cytocentrifuge (model 2 Cytospin; Shandon Scientific Co., Pittsburg, PA), and differential cell counts were performed using the centrifuged preparations stained with Diff-quick, counting 500 or more cells for each animal at a magnification of ×1000 (oil immersion).

### Cytokine measurement

The levels of IL-4 and IFN-γ were quantified in the supernatants of BAL fluids by enzyme immunoassays according to the manufacturer's protocol (Endogen Inc., Woburn, MA). The sensitivity of the assays was 5 pg/ml.

### Preparation of lung tissues and morphological analysis

The mice were euthanized after the final exposure, and the lungs and trachea were filled intratracheally with a fixative (0.8% formalin, 4% acetic acid) using a ligature around the trachea. The lungs were removed, and lung tissues were fixed with 10% (vol/vol) neutral buffered formalin. The specimens were dehydrated and embedded in paraffin. For histological examination, 4-μm sections of fixed, embedded tissues were cut on a Leica model 2165 rotary microtome (Leica Microsystems, Nussloch, Germany), placed on glass slides, deparaffinized, and stained sequentially with toluidine blue (Richard-Allan Scientific, Kalamazoo, MI). Selected toluidine blue-stained sections were used for measuring epithelial, goblet, and smooth muscle cells providing that the epithelium and submucosa could be easily identified and that the number of epithelial, goblet, and smooth muscle was adequate to allow multiple measurements (i.e., approximately 1 mm). Areas of the lung tissue with intact surface epithelium were selected for examination and quantification under a transmission electron microscope (H-7000; Hitachi, Tokyo, Japan). Ultrathin sections were cut, placed on high-transmission, 200-mesh, thin-bar copper grids, and stained with uranyl acetate and lead citrate. Light microscopic quantification was performed at ×200, and electron microscopy was performed at ×5000.

The cells that were counted (e.g., myofibroblasts) were used as evidence of airway remodeling rather than inflammation in a subepithelial zone of the entire transmission electron microscopy section, and the counts were expressed per 0.1 mm^2 ^of tissue. Myofibroblasts were identified by spindle-like projections, dilated rough endoplasmic reticulum, a greatly infolded and crenated nuclear membrane, and bundles of parallel cytoplasmic filaments associated with dense body condensations. The sections were coded and examined under light microscopy in random order by the same observer, who was unaware of the origin of the sections. Intra-observer repeatability was assessed by measuring the same section four times and was expressed as a percentage of the coefficient of variation for the four measurements.

### Statistical analysis

All data were analyzed using SPSS version 7.5 for Windows (SPSS Inc., Chicago, IL). The data are expressed as means ± SE. For measured variables with a normal distribution, Student's paired *t*-test was used to compare paired data. For variables that did not have a normal distribution, the Mann-Whitney *U*-test was used for comparisons. Differences with *p*-values less than 5% were regarded as statistically significant.

## Results

**Figure 2:**
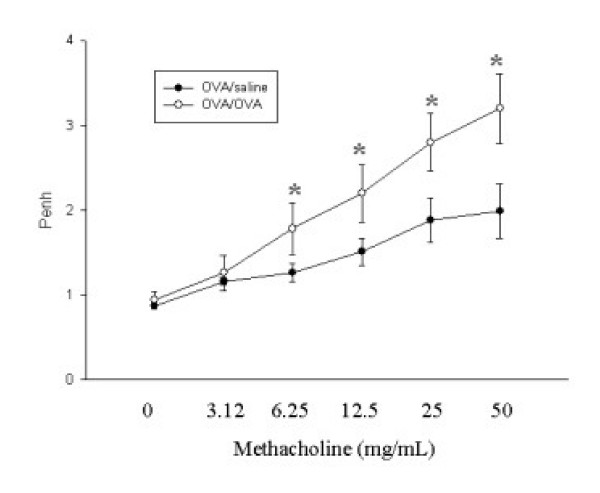
Methacholine-induced airway responses measured by whole-body plethysmography in BALB/c mice challenged with saline and ovalbumin. Values are means ± SE; *n *= 6 mice per group. * *p *< 0.05 compared to the group exposed to filtered air.

**Figure 3:**
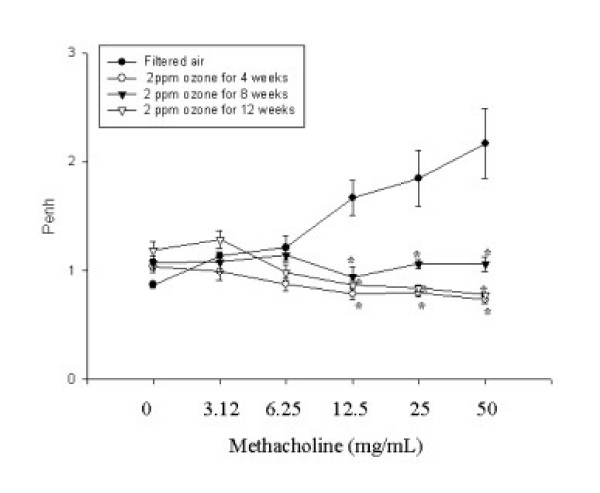
Methacholine-induced airway responses measured by whole-body plethysmography in BALB/c mice exposed to filtered air and to 2 ppm ozone for 8 h per day for 4, 8, and 12 weeks. Values are means ± SE; *n *= 6 mice per group. * *p *< 0.05 compared to the group exposed to filtered air.

The OVA-exposed group demonstrated significantly increased Penh at methacholine concentrations of 6.25, 12.5, 25, and 50 mg/ml compared to that of the saline-exposed group (Fig. 2). The ozone-exposed group demonstrated significantly decreased Penh at methacholine concentrations of 12.5, 25, 50 mg/ml compared to that of the filtered-air group (Fig. 3). We did not observe any differences in inflammatory cells or the levels of cytokines in the BAL fluids, or any changes in airway remodeling among the groups exposed to filtered air for 4, 8, and 12 weeks (data not shown). Therefore, we used the data for the group exposed to filtered air for 4 weeks in the comparisons to the ozone-exposed groups.

The proportions of eosinophils and neutrophils in BAL fluids were significantly higher in the group exposed to ozone for 4 weeks than in the filtered-air group (filtered-air group vs. ozone-exposed for 4 vs. 8 vs. 12 weeks: eosinophils, 1.5 ± 0.28 vs. 2.5 ± 0.13 vs. 1.11 ± 0.05 vs. 1.8 ± 0.08%; neutrophils, 2.2 ± 1.32 vs. 4.5 ± 1.02 vs. 1.9 ± 1.22 vs. 2.5 ± 2.01%, respectively; *p *< 0.05).

The INF-γ level decreased significantly after 4, 8, and 12 weeks of ozone exposure compared to that of the filtered-air group. The IL-4 level in BAL fluids was not different between any of the ozone-exposed groups and the filtered-air group (filtered-air group vs. ozone-exposed for 4 vs. 8 vs. 12 weeks: IFN-γ, 75.4 ± 2.57 vs. 30.3 ± 9.52 vs. 64.9 ± 2.9 vs. 55.6 ± 6.64 pg/ml; IL-4, 33.3 ± 3.27 vs. 65.1 ± 2.96 vs. 55.6 ± 6.64 vs. 45.9 ± 5.26 pg/ml, respectively). The ratio of IL-4 to INF-γ increased significantly after 4, 8, and 12 weeks of ozone exposure compared to the ratio of the filtered-air group (filtered-air group vs. ozone-exposed for 4 vs. 8 vs. 12 weeks: 0.43 ± 0.1 vs. 3.24 ± 3.4 vs. 1.30 ± 0.89 vs. 0.96 ± 0.38, respectively; *p *< 0.05). The ozone-exposed groups also demonstrated significantly increased protein levels compared to that of the filtered-air group (filtered-air group vs. ozone-exposed for 4 vs. 8 vs. 12 weeks: 10.07 ± 0.06 vs. 14.55 ± 0.76 vs. 11.12 ± 0.03 vs. 12.05 ± 0.11 μg/μl; *p *< 0.01).

**Figure 4:**
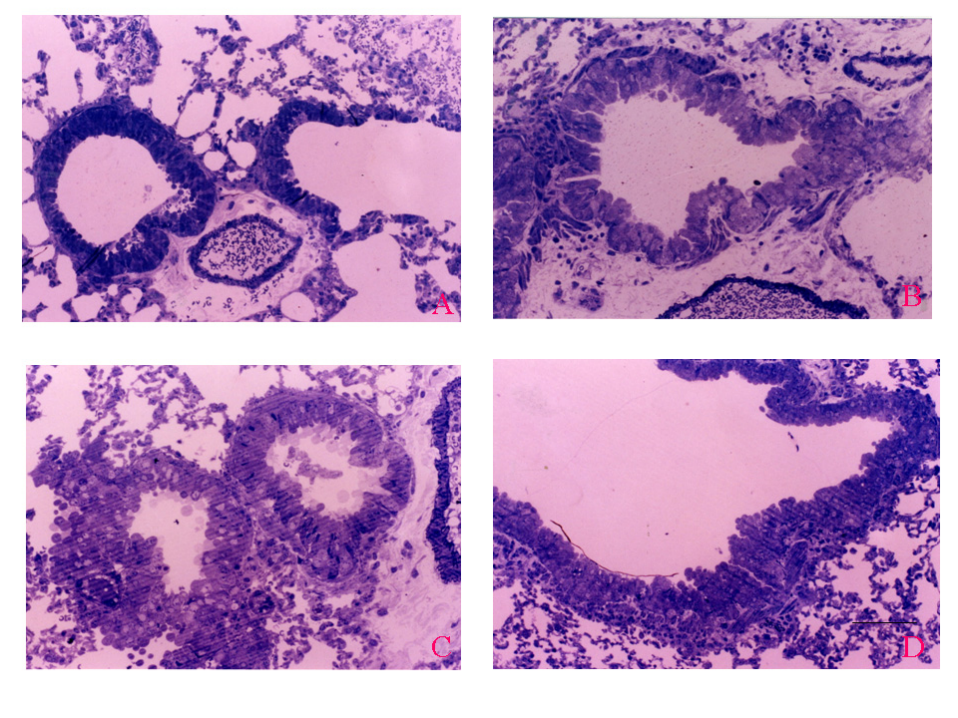
(A) Bronchioles exposed to filtered air have normal-appearing bronchioles and bronchiolo-alveolar portal and a normal transition from the low columnar epithelium lining the terminal bronchioles to the attenuated epithelium lining the alveoli. (B–D) Bronchioles exposed to 2 ppm ozone for 4, 8, and 12 weeks. (B) Pseudostratified bronchiolar epithelium and goblet cell metaplasia. (C)Markedly increased number of goblet cells. (D) Peribronchiolar collagen deposition and thickened smooth muscle cell coat. Original magnification, ×200. Scale bar = 100 μm.

**Figure 5:**
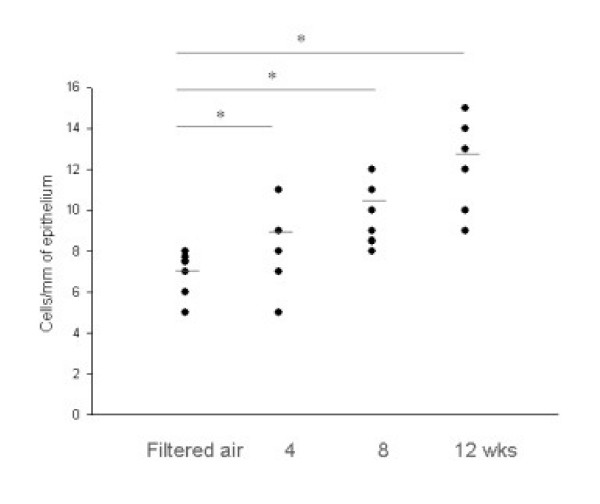
Goblet cell counts in the epithelium of bronchioles of mice exposed to filtered air and to 2 ppm ozone for 4, 8, and 12 weeks. The results are expressed as number of cells per millimeter of basement membrane. Horizontal bars represent median values. * *p *< 0.05 compared to the group exposed to filtered air.

**Figure 6:**
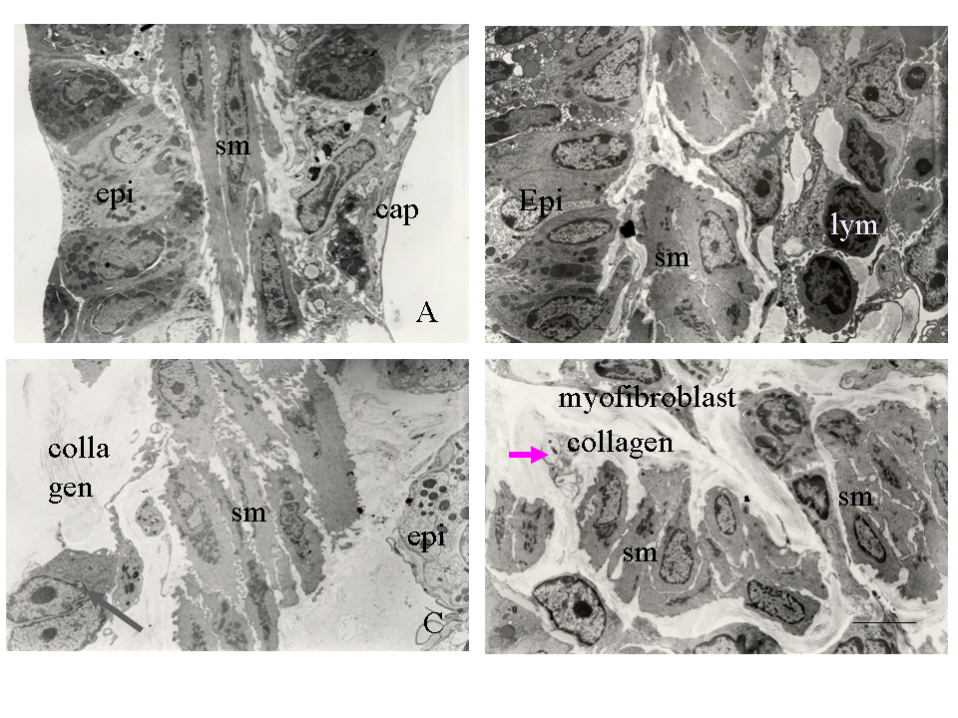
(A) Transmission electron micrograph of a bronchiole specimen from mice exposed to filtered air showing normal epithelium, smooth muscle, and capillaries. (B–D) Transmission electron micrographs of bronchiole specimens from mice exposed to 2 ppm ozone for 4, 8, and 12 weeks showing (B) hypertrophied smooth muscle cells (sm), a few infiltrating lymphocytes (lym), and myofibroblasts (arrow); (C) myofibroblasts (arrow), interstitial deposition of collagen fibers, and increased smooth muscle cell hypertrophy; and (D) disorganized smooth muscle cells, increased deposition of collagen fiber, unmyelinated nerve fiber (arrow), and myofibroblasts. Original magnification, ×5000. Scale bar = 5 μm.

**Figure 7:**
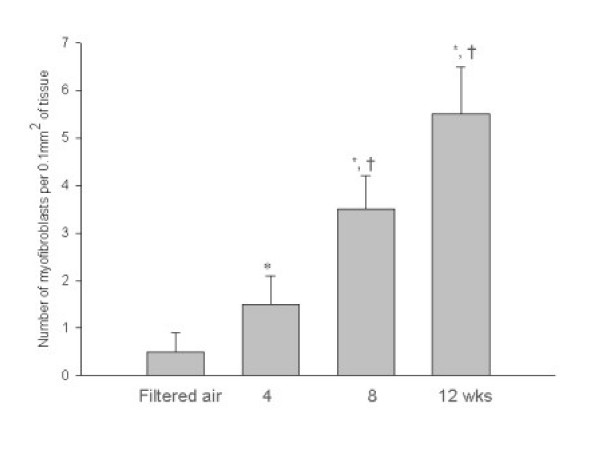
Mean number of myofibroblasts counted under electron microscopy in the subepithelial zone of specimens from mice exposed to filtered air and to 2 ppm ozone for 4, 8, and 12 weeks. The myofibroblasts comprised cells with elongated projections, dilated rough endoplasmic reticulum, an infolded or crenated nuclear membrane, and bundles of parallel cytoplasmic filaments associated with dense-body condensations. * *p *< 0.05 compared to the group exposed to filtered air. † *p *< 0.05 compared to the group exposed to ozone for 4 weeks.

The development of airway remodeling in the lungs of ozone-exposed mice was assessed by histological examination of toluidine blue-stained sections of lung tissue. The lungs of mice exposed to ozone for 4, 8, and 12 weeks were isolated, and representative 5-μm paraffin sections of lung tissue (3× sections every 100 μm) were examined. The number of goblet cells was significantly greater in the airway epithelium of mice after 4, 8, and 12 weeks of chronic exposure to ozone than after exposure to filtered air (Fig. 4). In addition to the marked increase in goblet cell number, an increased peribronchiolar collagen layer and a thickened smooth muscle coat were observed in the lung tissue sections from the ozone-exposed groups (Figs. 4 and 5; *p *< 0.05). Electron microscopic observations revealed increased collagen fiber deposition, increased smooth muscle cell hypertrophy and hyperplasia, and smooth muscle cell disorganization in the lung tissue sections from the ozone-exposed groups (Fig. 6). The number of myofibroblasts significantly increased in the subepithelial zone after 4, 8, and 12 weeks of chronic exposure to ozone compared to the number in mice exposed to filtered air (Fig. 7; *p *< 0.05).

## Discussion

We examined the effects of long-term exposure to ozone on airway remodeling and dysfunction in a mouse model of allergic airway disease. By measuring airway responses to methacholine, we found a decrease in AHR after long-term ozone exposure. We also observed collagen deposition and smooth muscle cell hyperplasia and hypertrophy in mice subjected to long-term ozone exposure. These changes suggest that chronic airway remodeling may be associated with AHR and airway inflammation following long-term exposure to ozone.

Chronic but not brief allergen exposure was associated with a markedly increased amount of extracellular matrix in the subepithelial region of the airway wall and with increased mucin content within the airway epithelium at 4 and 8 weeks after the last allergen challenge [20]. Repeated inflammatory events may contribute to airway remodeling in asthma [21]. Animal studies of allergen-induced AHR have shown that prolonged OVA exposure results in the increased deposition of fibronectin and collagen, which was accompanied by a progressive decrease in AHR, indicating that thickening or stiffening of the airway may be protective against AHR [22,23]. The increase in goblet cell number and in mucus lining the airway may serve a protective function against inhaled toxins and excessive mucosal dehydration [24].

We measured lung function using unrestrained plethysmography, which in conscious mice represents the extreme of noninvasiveness and is highly convenient; however, it provides respiratory measurements that are so tenuously linked to respiratory mechanics that they cannot be considered as meaningful indicators of lung function [25]. In our study using a murine model of asthma, the increase in AHR following OVA sensitization and challenge decreased after repeated exposure to ozone over a period of up to 12 weeks, indicating that structural airway changes can occur as protection against AHR after repeated exposure to air pollutants. Such changes included goblet cell hyperplasia, increased myofibroblast proliferation, increased collagen deposition, and smooth muscle hypertrophy and hyperplasia. Many asthma patients present evidence of residual airway obstruction, which can exist in asymptomatic patients, after antiasthma drugs; this probably represents remodeling. Remodeling may also be important in the pathogenesis of nonspecific AHR, especially the component that reverses slowly or incompletely with inhaled glucocorticosteroid treatment [26].

Airway injury or inflammation caused by air pollutants has been evaluated mainly by the analysis of fluids collected by bronchoalveolar lavage, which is an especially invasive technique totally unsuitable for children. Research in the field of biomarkers is providing new perspectives with the development of noninvasive tests for monitoring inflammation and damage in the deep lung. Our data in a murine model of asthma suggest that repeated exposure to air pollutants can induce airway remodeling and may account for irreversible airway obstruction.

It is necessary to speculate on how various aspects of the remodeling process could contribute to airway dysfunction and nonspecific AHR. IL-4 produced by several cell types, predominantly by Th2 lymphocytes, is believed to contribute to the characteristic inflammatory response in asthmatic airways [27]. IL-4 can modulate the behavior of fibroblasts [13] and may stimulate fibroblast-mediated contraction of extracellular matrix, as in a model of the tissue remodeling characteristics of fibrotic lesions [28]. The Th2-derived cytokines, IL-4 and IL-13, can stimulate the production of TGF-β in airway epithelial cells but not in lung fibroblasts. IFN-γ, in contrast, can inhibit TGF-β2 release both under basal conditions and following IL-4 or IL-13 stimulation. The ability of these cytokines to modulate TGF-β release may contribute to both normal airway repair and the development of subepithelial fibrosis in asthma [29]. In the present study, the decrease in INF-γ, the trend toward an increase in IL-4, and the increase in the ratio of IL-4 to INF-γ after chronic ozone exposure may contribute to structural airway changes following repeated ozone exposure in a murine model of asthma. Further studies are needed to clarify the potential mechanisms responsible for the AHR decrease in ozone-exposed mice despite the increase in airway smooth muscle mass and airway inflammation, as shown in the present study.

In conclusion, we have demonstrated that the airway physiology and airway structure are altered in a murine model of asthma chronically exposed to ozone. Sustained airway dysfunction was observed after 4 weeks of ozone exposure, and airway remodeling was sustained following 12 weeks of ozone exposure. The observation that airway remodeling persists after the recovery of AHR supports the postulate that structural changes contribute to changes of AHR in mice chronically exposed to ozone.
